# ABO Blood Grouping and Rhesus Factor Determination From Dental Pulp Tissue: A Forensic Research

**DOI:** 10.7759/cureus.43386

**Published:** 2023-08-12

**Authors:** Moumalini Das, Abhishek Banerjee, Jaydeep Samanta, Banga Bhusan Bhunia, Samaresh Mozumder, Karthikeyan Ramalingam

**Affiliations:** 1 Oral and Maxillofacial Pathology, Awadh Dental College and Hospital, Jamshedpur, IND; 2 Orthodontics and Dentofacial Orthopedics, Haldia Institute of Dental Sciences and Research, Haldia, IND; 3 Oral Pathology and Microbiology, Saveetha Dental College and Hospitals, Saveetha Institute of Medical and Technical Sciences, Saveetha University, Chennai, IND

**Keywords:** dental pulp, forensic odontology, absorption-elution technique, rh factor, abo blood grouping

## Abstract

Introduction

Collection of blood samples from mass disaster victims and blood grouping is a challenging task. This can be attributed to various reasons. From the forensic odontology perspective, teeth and bones are one of the noteworthy remains that can be derived from such sites.

Aims and objectives

The aim of our study is to detect ABO blood groups and Rhesus (Rh) factor from extirpated pulp tissue of the extracted teeth at zero, three, and six months’ time interval by absorption-elution technique.

Materials and methods

The study consisted of 90 freshly extracted teeth as suggested by a biostatistician. Thirty teeth were analyzed immediately and 60 teeth were stored in vials without any preservative at room temperature. The pulp tissue was extirpated and studied at zero months, three months, and six months to determine blood groups and Rh factors. The extraction socket blood was tested to identify the blood group of that patient and used as a control reference. The blood grouping was done at respective time periods through the absorption-elution method and matched with the control. Data were analyzed using Statistical Package for the Social Sciences (SPSS) version 24 software (IBM Corp., Armonk, NY). The chi-square test and Kruskal-Wallis test were done. A p-value < 0.05 was considered statistically significant.

Results

Pulp showed the highest sensitivity for blood groups at zero months but it could be identified up to six months, although the sensitivity and specificity gradually decreased. ABO blood grouping showed higher sensitivity than the Rh factor as time progressed.

Conclusion

In cases where teeth are the only remains in a forensic condition, the dental pulp can be an authentic source for blood group detection.

## Introduction

Forensic odontology, forensic dentistry, or forensic odontostomatology, according to the Federation Dentaire Internationale, can be defined as “a branch of dentistry which deals with the proper handling and examination of dental evidence and with the proper evaluation and presentation of dental findings in the interest of justice” [1,2]. Dr. Oscar Amoedo is the father of forensic odontology. He made the identification of victims of a fire accident in Paris (1897) [2,3].

Disasters are circumstances that are unpredictable and have destruction potential of unforeseen immensity. Disaster victim identification (DVI) after destruction comprises several techniques and systems having a role in identifying expired victims in mass devastation [3]. DVI can be categorized into five sequences of activity, namely, the scene, mortuary, collection of ante-mortem data, reconciliation of data, and debriefing [1,3]. Maximum countries include forensic odontologists in the DVI squad. This group consists of people having multifaceted talents [3,4]. A doctor’s performance as a forensic odontologist goes along with the police officers in demonstrating the “IDENTITY” of a person affected by mass devastations [4,5].

Dental characteristics are one of the very important ways for the establishment of identification of victims in cases of forensic investigations. The dental aspect gives much information in comparison with other body structures that can help in forensic identification [6,7]. Teeth intrinsically possess a very resistant nature. They are well preserved even in highly adverse environmental conditions and have a major role as mortal remains for identification [6,8].

The blood group substances can be used in medicolegal examination and it is one the keystone for the establishment of identity of biological materials in forensic odontology. It is because once a blood group of an individual is established, it remains constant throughout his life [9]. Similar to fingerprinting, the blood group of an individual is unchangeable in nature [10]. Karl Landsteiner, in the year 1900, first described the ABO blood group system [11,12]. In the year 1960, Kind discovered the ABO blood group in saliva by a specific method called the “absorption-elution method.” This technique is now employed in all forensic laboratories, as it is the most reliable, sensitive, and reproducible method [6,12,13]. The pulp tissue is highly vascular; therefore, the antigens of the blood group are obviously present in the pulp. The dental hard tissues provide good protection to the pulp from any harmful effects of trauma, heat, or any outer influence. So very lately, post-mortem degradation can also be noted in them [6,8,14].

ABO blood group determination is very important for identifying a deceased person in every mass disaster and also has a role in clinical medicine and human genetics. Therefore, dental structures, particularly pulp, are a great resource for these purposes where only a tooth remains after any mass devastation and thus it is considered very valuable. It is of immense significance for forensic odontologists, as they play a major role in every forensic investigation.

In our Indian scenario, forensic odontology is a growing and challenging field that needs a lot of research work and more published data, as there are extensive regional and ethnic variations among the Indian population. The aim of our study was to analyze the blood groups from pulp samples of extracted teeth at varying time intervals.

We did this study for the detection of ABO blood groups and Rhesus (Rh) factor from extirpated pulp tissue of the freshly extracted teeth done at three months intervals till six months (zero months - instantly, three months, and six months) interval by absorption-elution technique. To the best of our knowledge, a forensic study was not done previously in these tribal areas or any districts of Jharkhand. We also analyzed the pulp tissue yield (in cc) in measurements of volume for each tooth with increasing time periods.

## Materials and methods

The patients visiting the outpatient department of Awadh Dental College and Hospital, Jamshedpur (East Singhbhum district) for extraction from January 2023 till June 2023 were included in the study. An institutional ethical clearance certificate was obtained from the Institutional Ethical Committee, Awadh Dental College and Hospital (letter number: ADCH/EC/OP/212409698295).

Prior written informed consent was obtained from the patients involved in the study. A total of 90 freshly extracted teeth were collected within the age group of 20-50 years. Out of them, 30 teeth were instantly subjected to pulp extirpation at the laboratory, while the other 60 teeth were stored in vials without any preservative for further study.

Inclusion criteria were teeth indicated for extraction among patients exclusively of tribal origin residing in the vicinity of Dalma Hills of East Singhbhum district of Jharkhand. Orthodontic extractions, mobile vital teeth, and impacted teeth were included. Preferences were given to incisors, canines, premolars, and molars.

Exclusion criteria were non-vital teeth, endodontically treated teeth, and grossly destroyed teeth indicated for extraction. Patients not belonging to the tribal region in the vicinity of Dalma Hills and patients aged less than 19 years or older than 51 years of age were excluded from the study.

Method of blood grouping of the patients

A detailed case history was recorded for included patients prior to the collection of teeth and blood from their extraction sockets. The blood from the extraction socket of the patient undergoing extraction was aspirated with a syringe and collected into EDTA (ethylenediaminetetraacetic acid) vials along with the tooth extracted so that the blood did not clot till analysis. Blood grouping was performed by slide agglutination method and it was considered as a control reference for ABO blood groups/Rh factor, detailed case history of the patients was recorded, and informed consent was obtained from the patients along with ethical clearance.

The extracted tooth was rinsed with normal water three times for the removal of debris. Then it was wiped with a sterile gauze piece and was air-dried and thereafter it was kept in a vial. The blood group and date of extraction were mentioned in the vials for future reference. In this process, all the extracted teeth were collected and blood groups were ascertained and stored in vials without any preservative at room temperature. They were used for experiments at immediate analysis (zero months), three months, and six months.

Pulp extirpation method

**Figure 1:**
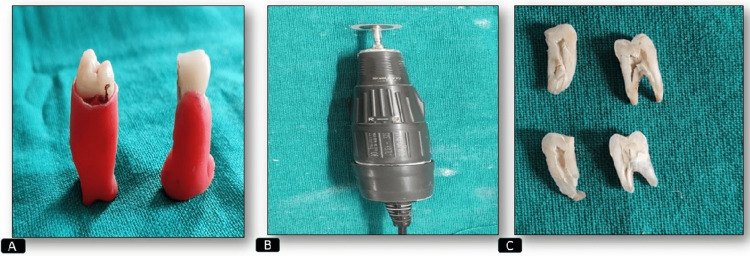
Picture showing the mounting and sectioning of teeth (A) Teeth embedded in modeling wax. (B) Micromotor attached with carborundum disc. (C) Teeth sectioned longitudinally with micromotor into equal halves for pulp extirpation with a spoon excavator.

Extracted tooth was embedded in a modeling wax block and sectioned using micromotor and carborundum disc longitudinally, following which the pulp was extirpated using a sterile spoon excavator (Figure 1).

The extirpated pulp was analyzed. It took around seven hours to carefully section the tooth, extirpate the pulp, and perform the ABO testing. Hence, we analyzed five to seven teeth per day to maintain accuracy and avoid any errors.

**Figure 2:**
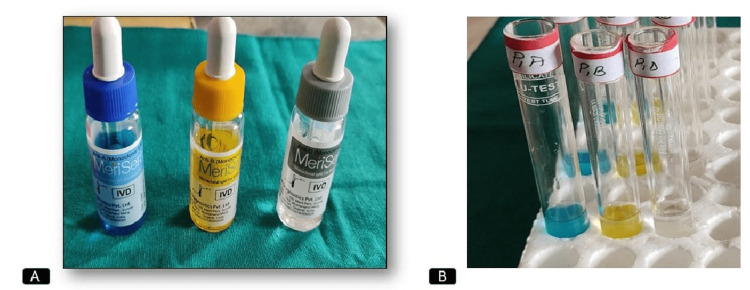
Picture of ABO reagents (A) Antiserum A, B, and D for the purpose of slide agglutination from extraction socket blood and experiment of ABO blood grouping and Rh factor grouping from the pulp. (B) Three drops of antiserum A, B, and D were added to three test tubes, respectively, containing pulp samples and kept soaked for 2 ½ hours at room temperature.

Test for ABO blood grouping and Rh factor from the dental pulp was done by absorption-elution technique. Each time for seven teeth during the experiment, test tubes were labeled as P1, P2, P3, P4, P5, P6, and P7. Three test tubes were required for each tooth, which were labeled as PA, PB, and PO. For example, for P1 teeth, we labeled P1A, P1B, and P1O in three test tubes in the same way. Labeling was done for reference and documentation purposes. Here, P denotes pulp samples and A, B, and O denote the type of antiserum that had to be given to the test tube and also the type of 0.5% red cell suspension of known blood group (A+, B+, and O+) that has to be added later (Figure 2).

**Figure 3:**
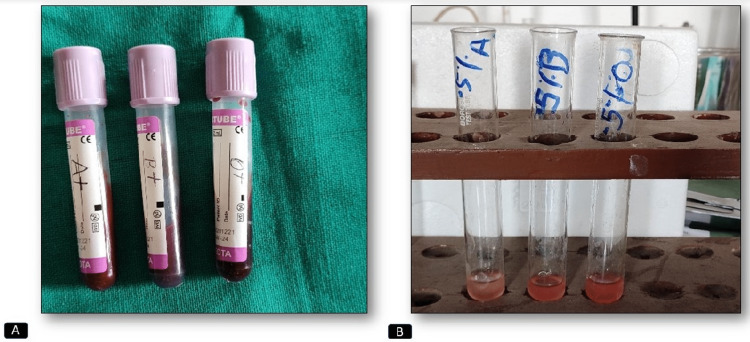
Picture showing ABO estimation (A) Ethylenediaminetetraacetic acid (EDTA) vials containing 1 ml of blood from individuals of known blood groups A+, B+, and O+ and labeled accordingly. (B) 0.5% A, 0.5% B, and 0.5% O red cell suspensions of known blood groups (A+, B+, and O+) were freshly prepared.

Three individuals with known blood groups (A+, B+, and O+) were chosen and 1 ml of venous blood was taken in a syringe from each one of them separately. Prior to this procedure, consent was taken from them. Then, blood was transferred to EDTA vials separately so that it did not clot (Figure 3). Then blood was transferred to separate test tubes and was labeled as A+, B+, and O+, respectively. These blood samples were centrifuged for one minute at 1000 rpm. The plasma collected on top after centrifugation was discarded using a separate micropipette. Then saline was taken up to 3/4th of the test tube and centrifuged; thereafter, the supernatant was again discarded. This is referred to as cell washing. This process of cell washing was repeated two times or more until the normal saline solution was clear. Now, three separate test tubes were taken and named 0.5% A, 0.5% B, and 0.5% O, respectively. Two milliliters of saline was added to those labeled test tubes with a pipette, and from the prepared RBC test tubes, 10 µl was taken into a micropipette and added to them, using a separate micropipette each time. Thus, the 0.5% red cell suspension was ready to use and kept in an upright manner, namely, 0.5% A, 0.5% B, and 0.5% O red cell suspension, respectively (Figure 3).

**Figure 4:**
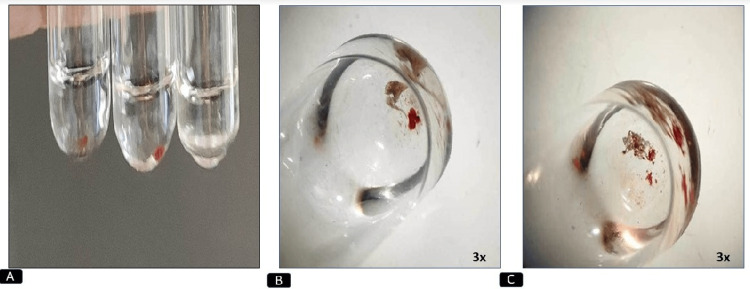
Analysis of agglutination (A) Macroscopic observation of agglutination of pulp samples in three test tubes. (B, C) Stereo microscopic images of agglutination of pulp samples in a test tube.

Agglutination at the end of the experiment was observed both macroscopically (Figure 4) and under the stereomicroscope (Figure 4).

**Figure 5:**
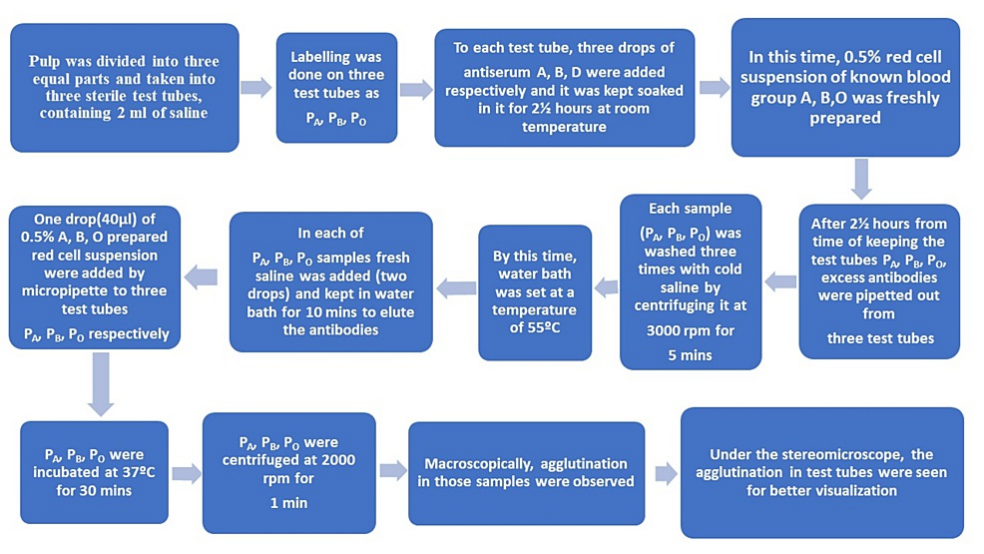
Flowchart Flowchart describing the process for the test for ABO blood grouping and Rh factor from the dental pulp by absorption-elution technique.

A flowchart has been formulated for the process (Figure 5).

The blood group obtained after an experiment from the pulp was finally matched with the control.

Statistical analysis

The data were tabulated in Microsoft Excel (Microsoft Corporation, Redmond, WA) and analyzed using Statistical Package for the Social Sciences (SPSS) version 24 software (IBM Corp., Armonk, NY). The variables are presented with frequency and percentage. The chi-square test and Kruskal-Wallis test were used for the comparisons. A p-value ≤ 0.05 was considered statistically significant.

## Results

ABO blood group determination from the pulp of 30 patients at zero months, 30 patients at three months, and 30 patients at six months is tabulated in Table 1, where the number (N) and percentage (%) of matched and unmatched blood groups are shown.

**Table 1:** ABO analysis Table showing the ABO blood group determination from the pulp at zero months (30 patients), three months (30 patients), and six months (30 patients).

Time period	Blood group matched		Blood group unmatched	
	N	%	N	%
0 months	26	86.67	4	13.33
3 months	23	76.67	7	23.33
6 months	10	33.33	20	66.67

Similarly, Rh factor grouping from the pulp of the same 30 patients at zero months, 30 patients at three months, and 30 patients at six months is tabulated in Table 2, where the number (N) and percentage (%) of matched and unmatched blood groups are shown.

**Table 2:** Rhesus (Rh) factor estimation Table showing the Rh factor estimation from the pulp at zero months (30 patients), three months (30 patients), and six months (30 patients).

Time period	Rh factor matched		Rh factor unmatched	
	N	%	N	%
0 months	26	86.67	4	13.33
3 months	23	76.67	7	23.33
6 months	8	26.67	22	73.33

**Figure 6:**
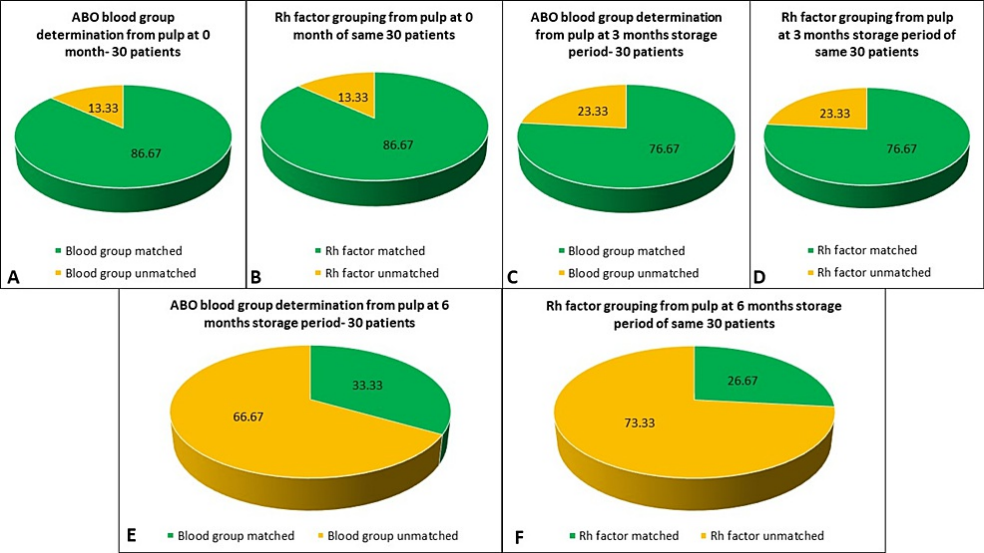
Pie diagram (A) ABO blood group determination from the pulp at zero months (30 patients). (B) Rhesus (Rh) factor grouping from the pulp at zero months of the same 30 patients. (C) ABO blood group determination from the pulp at three months storage period (30 patients). (D) Rh factor grouping from the pulp at three months storage period of the same 30 patients. (E) ABO blood group determination from the pulp at six months storage period (30 patients). (F) Rh factor grouping from the pulp at six months storage period of the same 30 patients.

The above two tables are shown in the form of individual pie charts for ABO blood grouping and Rh factor grouping in Figure 6.

A table representing the distribution of the ABO blood groups and Rh factor is shown in Table 3.

**Table 3:** Study outcome Table showing the distribution of the ABO blood groups and Rh factor in our study.

Blood groups	N	%
A+	13	14.44
A-	2	2.22
B+	21	23.33
B-	4	4.44
AB+	21	23.33
AB-	4	4.44
O+	18	20
O-	7	7.77
	90	99.97

**Figure 7:**
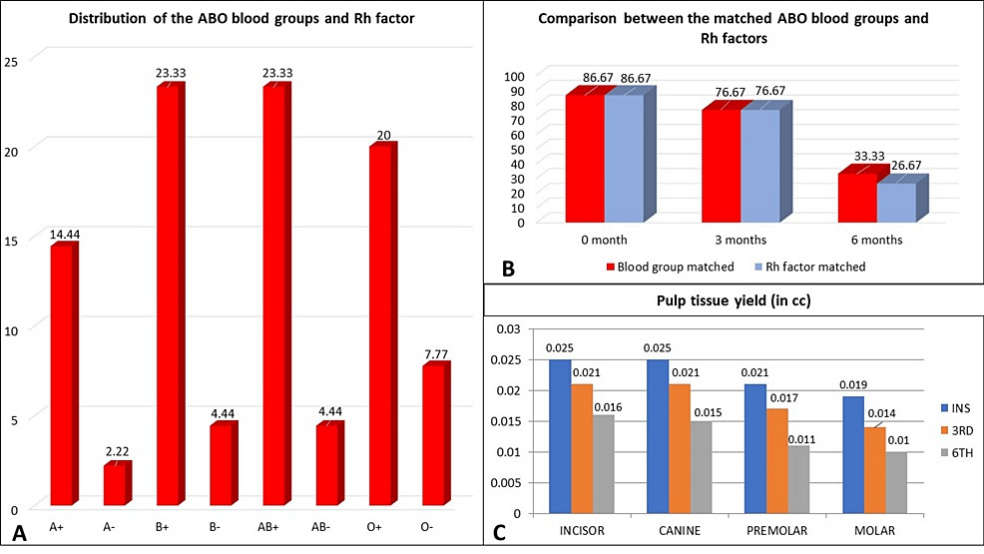
Graphs (A) Distribution of the ABO blood groups and Rh factors. (B) Comparison between the matched ABO blood groups and Rh factors. (C) Pulp tissue yield in cc from teeth in measurements of the volume of each tooth such as incisor, canine, premolar, and molar at zero (instant), three, and six months.

According to these data, B+ and AB+ were found in the maximum number whereas A- was found in the minimum number (Figure 7).

Graphs representing the comparison between the matched ABO blood groups and Rh factors of pulp and extraction socket blood at zero months, three months, and six months are shown in Table 4. P-value came to be statistically insignificant. In all the graphs, N denoted the number of patients.

**Table 4:** ABO and Rhesus (Rh) factor comparison Table showing individual A+, A-, B+, B-, AB+, AB-, O+, and O- blood group comparisons at zero months, three months, and six months.

Blood groups	0 months (n = 30)	3 months (n = 30)	6 months (n = 30)	N
A+	5	5	3	13
A-	1	1	0	2
B+	4	9	8	21
B-	3	0	1	4
AB+	8	5	8	21
AB-	1	2	1	4
O+	7	7	4	18
O-	1	1	5	7
Number of patients (N)	30	30	30	90

Pulp tissue yield (in cc) from teeth in measurements of the volume of each tooth like incisor, canine, premolar, and molar at zero, three, and six months is tabulated in Table 5.

**Table 5:** Pulp tissue yield Table showing pulp tissue yield (in cc) from various teeth. I = incisor; C = canine; P = premolar; M = molar.

Time period	Type of teeth	Number of teeth	Volume of pulp in cc (approx.)/tooth
0 months	I = 8, C = 6, P = 8, M = 8	30	I = 0.025
			C = 0.025
			P = 0.021
			M = 0.019
3 months	I = 10, C = 2, P = 8, M = 10	30	I = 0.021
			C = 0.021
			P = 0.017
			M = 0.014
6 months	I = 9, C = 7, P = 6, M = 8	30	I = 0.016
			C = 0.015
			P = 0.011
			M = 0.010

It was seen that with time, the pulp tissue yield from teeth was decreased (Table 6). The Kruskal-Wallis test was done and the chi-square values were obtained. Pulp tissue yield (in cc) in the measurement of volume for incisor, canine, premolar, and molar was decreased over time from zero to six months and it was found to be statistically significant (p < 0.05). Moreover, orthodontic and non-orthodontic teeth at zero, three, and six months did not show any differences in pulp tissue yield.

**Table 6:** Kruskal-Wallis test Table showing the statistical analysis for pulp tissue yield (in cc) over increasing time periods for all teeth.

Type of teeth	Time period	N (number of teeth)	Mean rank	Chi-square value	Asymptomatic significant (p)
Incisor	0 months	8	23.50	26.000	0.000
	3 months	10	14.50		
	6 months	9	5.00		
Canine	0 months	6	12.50	14.000	0.001
	3 months	2	8.50		
	6 months	7	4.00		
Premolar	0 months	8	18.50	21.000	0.000
	3 months	8	10.50		
	6 months	6	3.50		
Molar	0 months	8	22.50	25.000	0.000
	3 months	10	13.50		
	6 months	8	4.50		

## Discussion

Identification of dental structures is a prompt, fastest, economical, and uncomplicated technique for establishing the identity of an individual. Its success is rational with data obtained from other procedures [3,9,11].

Overall distribution of blood groups as per our study of East Singhbhum district of Jharkhand in tribal population of Dalma Hills for the ABO blood groups and Rh factor was A+ (14.44%), A- (2.22%), B+ (23.33%), B- (4.44%), AB+ (23.33%), AB- (4.44%), O+ (20.00%), and O- (7.77%). B+ and AB+ were found in the maximum number whereas A- was found in the minimum number. These percentages were calculated from our study.

Pulp tissue is extremely vascular and it contains the blood group antigens apart from the endothelial cells and RBCs [11,15]. There may be some other methods but they are not reliable. The absorption-elution technique is the most sensitive and specific method and is therefore used for the detection of blood groups. Pulp tissue shows high specificity for detecting both ABO blood groups and Rh factors [16-18].

The tribal population mainly stays in the vicinity of the Dalma Hills of East Singhbhum district of Jharkhand. The biggest tribal community is Santal or Santhal. The tribal population census of East Singhbhum district is 27.8% with 2000-6000 people residing in Dalma Hills. It is a remote and neglected area. Very few people from these tribal regions came to the outpatient department of our dental college and we have tried to decipher their blood groups with dental samples.

At zero months, out of 30 teeth, 26 teeth gave positive findings. The sensitivity of both the ABO blood group and Rh factor was very high at zero months. At the three-month time interval, the sensitivity slightly decreased for both ABO blood groups and Rh factor. Out of 30 teeth, 23 gave positive results. Sensitivity decreased further for both at a six-month time interval. Out of 30 teeth studied, 10 teeth gave positive results for ABO blood groups and eight teeth for Rh factor.

It can be seen that ABO blood grouping sensitivity and specificity were equal at zero months and three months but the sensitivity of ABO blood grouping was somewhat better than Rh factor at six months. The blood group was matched with the control for up to six months but the antigenicity of the pulp was decreasing and thus the number of matched blood groups decreased. It is hereby understood that the sensitivity of the ABO blood group was slightly better than the Rh grouping at six months.

In a total of 90 teeth, 59 teeth matched with control for ABO blood grouping, and 57 teeth matched with control for Rh factor. Overall, it was found that the sensitivity of ABO blood groups was better than Rh factor grouping up to six months. Observations from our study also showed that there were no problems in yielding pulp tissue from teeth between orthodontic and non-orthodontic teeth extractions and the volume of pulp tissue yield was the same in any of the types of extractions.

This study was consistent with Kumar et al., who studied 150 cases of extracted teeth and estimated the blood groups from pulp and dentin. The sensitivity of ABO blood groups was greater than the Rh factor and the p-value was statistically insignificant at six months, similar to our study [11]. The study conducted by Ramnarayan et al. showed a sensitivity of 83.3% in pulp and it showed a high correlation in freshly extracted teeth and less correlation in six months of storage time of teeth. It was in accordance with our study [10]. The study conducted by Vala et al. was also in unison with our study where they followed the absorption-elution technique and studied for blood groups both from pulp and dentin, and pulp gave 80% positive results in freshly extracted teeth [7]. Saxena et al. also had similar results where they found the sensitivity of the pulp was 80% [4]. Sood et al. also experimented with ABO blood groups and Rh factor and found that 89 out of 100 teeth showed positive results after 180 days of extraction [14]. Rajawat et al. studied salivary samples in 300 individuals for ABO blood groups by the absorption-inhibition method and found that 250/300 were secretors. They concluded that blood groups could be identified specifically from the saliva of those who were secretors of antigen [18].

In our study, it could be clearly seen as the time period was increasing, antigenicity was decreasing and it had the tendency to show negative results at six months' time. Observation from our study indicated that dental pulp is a very good source to detect ABO blood grouping and Rh factor, as the pulp is the most vascular and cellular component of teeth. ABO blood grouping had higher sensitivity than Rh factor grouping and gave slightly higher positive results as time progressed. At six months, ABO blood group detection was better than Rh factor grouping.

The loss of antigenicity as the time period increased may be due to drying of the tissue, loss of antigens of pulp, inadequate pulp for the experiment, calcification, lysis of the pulp cells, putrefaction of the bodies in an actual forensic scenario, and bacterial contamination [18], and also aerobic gram-negative bacteria grew over a storage period and there is a possibility of some mistyping of blood groups. Some negative results may also be due to processing errors [11,14,17,18].

Our study is unique in that both ABO blood grouping and Rh factor grouping were done at zero, three, and six-month time intervals from the pulp of extracted teeth exclusively in people of tribal origin of East Singhbhum district of Jharkhand residing in the vicinity of the hills of Dalma. There is always an ethnic variation with evolutionary processes and anthropological changes can be seen. Moreover, in our study, the agglutination was finally visualized under a stereomicroscope for confirmation of agglutination and for better assessment. We have also tried to find out the amount of yield of pulp tissue in measurements of volume with time for each tooth and its variations among different types of teeth at zero, three, and six months.

Limitations

Our study mainly focused on dental pulp. Salivary expression of ABO antigens along with other hard tissue structures like enamel, dentin, cementum, and bone could be analyzed for their outcomes. Moreover, if the study is done for longer storage periods beyond six months, some additional information may be found.

## Conclusions

ABO blood grouping and Rh factor determination are very significant to identify a deceased person in a forensic investigation. In cases where teeth are the only remains in large-scale mass devastations, dental pulp plays a very vital role. Absorption-elution technique is employed to find the particular blood group of an individual with 100% accuracy. Thus, from our study, we can conclude that it is possible to detect ABO blood groups and Rh factors from dental pulp for up to six months. The blood group detection decreased with time, and the sensitivity of ABO blood groups is somewhat higher as compared to the Rh factor.
